# A Cotyledon-based Virus-Induced Gene Silencing (Cotyledon-VIGS) approach to study specialized metabolism in medicinal plants

**DOI:** 10.1186/s13007-024-01154-x

**Published:** 2024-02-12

**Authors:** Yongliang Liu, Ruiqing Lyu, Joshua J. Singleton, Barunava Patra, Sitakanta Pattanaik, Ling Yuan

**Affiliations:** Department of Plant and Soil Sciences and Kentucky Tobacco Research and Development Center, University of Kentucky, Lexington, KY 40546 USA

**Keywords:** Cotyledon-VIGS, *Catharanthus roseus*, *Glycyrrhiza inflata*, *Artemisia annua*

## Abstract

**Background:**

Virus-induced gene silencing (VIGS) is widely used in plant functional genomics. However, the efficiency of VIGS in young plantlets varies across plant species. Additionally, VIGS is not optimized for many plant species, especially medicinal plants that produce valuable specialized metabolites.

**Results:**

We evaluated the efficacy of five-day-old, etiolated seedlings of *Catharanthus roseus* (periwinkle) for VIGS. The seedlings were vacuum-infiltrated with *Agrobacterium tumefaciens* GV3101 cells carrying the tobacco rattle virus (TRV) vectors. The *protoporphyrin IX magnesium chelatase subunit H* (*ChlH*) gene, a key gene in chlorophyll biosynthesis, was used as the target for VIGS, and we observed yellow cotyledons 6 days after infiltration. As expected, the expression of *CrChlH* and the chlorophyll contents of the cotyledons were significantly decreased after VIGS. To validate the cotyledon based-VIGS method, we silenced the genes encoding several transcriptional regulators of the terpenoid indole alkaloid (TIA) biosynthesis in *C. roseus*, including two activators (*CrGATA1* and *CrMYC2*) and two repressors (*CrGBF1* and *CrGBF2*). Silencing *CrGATA1* led to downregulation of the vindoline pathway genes (*T3O*, *T3R*, and *DAT*) and decreased vindoline contents in cotyledons. Silencing *CrMYC2*, followed by elicitation with methyl jasmonate (MeJA), resulted in the downregulation of *ORCA2* and *ORCA3*. We also co-infiltrated *C. roseus* seedlings with TRV vectors that silence both *CrGBF1* and *CrGBF2* and overexpress *CrMYC2*, aiming to simultaneous silencing two repressors while overexpressing an activator. The simultaneous manipulation of repressors and activator resulted in significant upregulation of the TIA pathway genes. To demonstrate the broad application of the cotyledon-based VIGS method, we optimized the method for two other valuable medicinal plants, *Glycyrrhiza inflata* (licorice) and *Artemisia annua* (sweet wormwood). When TRV vectors carrying the fragments of the *ChlH* genes were infiltrated into the seedlings of these plants, we observed yellow cotyledons with decreased chlorophyll contents.

**Conclusions:**

The widely applicable cotyledon-based VIGS method is faster, more efficient, and easily accessible to additional treatments than the traditional VIGS method. It can be combined with transient gene overexpression to achieve simultaneous up- and down-regulation of desired genes in non-model plants. This method provides a powerful tool for functional genomics of medicinal plants, facilitating the discovery and production of valuable therapeutic compounds.

**Supplementary Information:**

The online version contains supplementary material available at 10.1186/s13007-024-01154-x.

## Background

Virus-induced gene silencing (VIGS) has emerged as an invaluable tool for post-transcriptional gene silencing in plants [1–3]. Compared to conventional genetic transformation methods, VIGS offers several advantages, including rapid implementation, efficiency, low cost, and independence of tissue culture and plant regeneration processes. VIGS is thus particularly useful for many non-model and recalcitrant plants [1, 2, 4]. Various RNA and DNA viruses have been employed in VIGS, and among them, tobacco rattle virus (TRV) is widely used due to its broad host range, efficient silencing outcomes, and mild symptoms on plants [3, 5–7]. TRV-based VIGS has been successfully applied in a wide range of plant species, including model plants such as *Arabidopsis thaliana* [8], *Nicotiana benthamiana* [6] and tomato (*Solanum lycopersicum*) [9], crops such as wheat (*Triticum aestivum*) and maize (*Zea mays*) [10], and medicinal plants such as *Catharanthus roseus* (Madagascar periwinkle) [11–16] and *Withania somnifera* (winter cherry) [17]. Moreover, TRV-based VIGS have been applied to different plant organs, including roots [18, 19], leaves [9], flowers [20], fruits [21], and seeds [22], making it a versatile tool for functional genomic research. Despite the many advantages of TRV-based VIGS, its broader application is limited by several factors, including variations in inoculation methods, as well as low and inconsistent efficiency in various plant species [23].

Agroinfiltration methods, through syringe or vacuum infiltration, are commonly used to transiently overexpress or knockdown a gene-of-interest. In syringe infiltration, a needle-free syringe carrying *Agrobacterium* suspension is placed on abaxial surface of leaf lamina and the suspension is slowly forced into leaves. Initially, the syringe infiltration method has been used to inoculate *Agrobacterium* carrying TRV vectors into the leaves of *N. benthamiana* [6]. However, this method was found unsuitable for some of the other plant species, leading to the development of diverse inoculation methods such as spray infiltration [9], vacuum infiltration [24], pinch wounding [11], Agrodrench [19], and sprout vacuum infiltration (SVI) [25]. In vacuum infiltration, the pressure differences between the surface and the inside of the leaf causes the penetration of *Agrobacterium* into the leaf’s intercellular space. Plant tissues immersed in *Agrobacterium* suspension is placed in a vacuum chamber. The pressure in the chamber is lowered for a short duration to release the air in the intercellular spaces through the stomata. The plant tissue is subjected to re-pressurization during which the suspension is drawn into the leaf through the stomata [26, 27]. For some plant species, determining the suitable inoculation method requires testing several different methods, which is time intensive. For example, four inoculation methods have been tested for *C. roseus*, and only the pinch wounding method is proven successful [11]. The SVI method has been optimized in four *Solanaceous* crops, including tomato, eggplant (*Solanum melongena*), pepper (*Capsicum annuum*), and *N. benthamiana*, and the method is faster than other inoculation methods, showing silencing phenotype in the first pair of true leaves [25]. However, the movement of the virus to the newly developed leaves, the efficiency and time vary in different plants. For instance, the efficiency of optimized SVI for two *Lycium barbarum* and *L. ruthenicum* (Goji) species only reaches approximately 30% [28]. Therefore, the development of a widely applicable and highly efficient method is necessary to advance VIGS technology.

*C. roseus* is a highly valued medicinal plant that accumulates almost 200 terpenoid indole alkaloids (TIAs), including the important anti-cancer drugs vinblastine and vincristine [29]. While the biosynthesis of TIAs in *C. roseus* has been extensively studied [30–32], efforts are still ongoing to better understand the regulatory mechanisms [33]. Methyl jasmonate (MeJA) is the major elicitor of TIA biosynthesis, and several transcription factors (TFs), such as CrMYC2 [34, 35], BISs [36–38], ORCAs [39–43], RMT1 [15] and CrGBFs [35, 44], have been characterized for their roles in the regulation of TIA biosynthesis in response to MeJA. The vindoline biosynthesis, which is not regulated by MeJA, is controlled by the GATA-type zinc-finger TF CrGATA1 [13]. To date, stable transformation to consistently generate transgenic *C. roseus* plants has been difficult. VIGS has been widely relied on by many laboratories in characterizing genes encoding biosynthetic enzymes, transporters, and regulators involved in TIA biosynthesis [13–15, 31, 45]. Because the complex, dimerized TIAs are synthesized in *C. roseus* leaves, transformation of hairy roots, although useful in genetic characterization, does not allow the investigation of TIA pathway genes that are predominantly expressed in the leaves. Like *C. roseus*, the generation of stable transgenic plants are difficult and time consuming for many other medicinal plants, such as *Glycyrrhiza inflata* [46] which produce the bioactive agent licochalcones. An efficient VIGS technique certainly benefits the studies of the biosynthesis and regulation of the specialized metabolites in these plants.

Here we describe the development of a cotyledon-based VIGS (cotyledon-VIGS) method for *C. roseus*, which is significantly faster and more efficient than the previously described pinch wounding method. We also successfully extended cotyledon-VIGS to medicinal plants *G. inflata* and *Artemisia annua*, indicating the broad applicability of the technique. Silencing *CrGATA1* or *CrMYC2* in *C. roseus* resulted in expected downregulation of their respective target genes and reduction in accumulation of TIAs. Additionally, we were able to silence two repressor *CrGBFs* and overexpress the activator *CrMYC2* simultaneously by combining cotyledon-VIGS with a transient gene overexpression method. Our findings demonstrated that cotyledon-VIGS is a versatile tool for analysis of gene functions in recalcitrant medicinal and crop plants. A protocol optimized for one plant species might work for other species; however, the parameters should still be optimized for each plant species to achieve the best results.

## Results

### Five-day-old *C. roseus* seedlings are ideal for cotyledon-VIGS

**Fig. 1:**
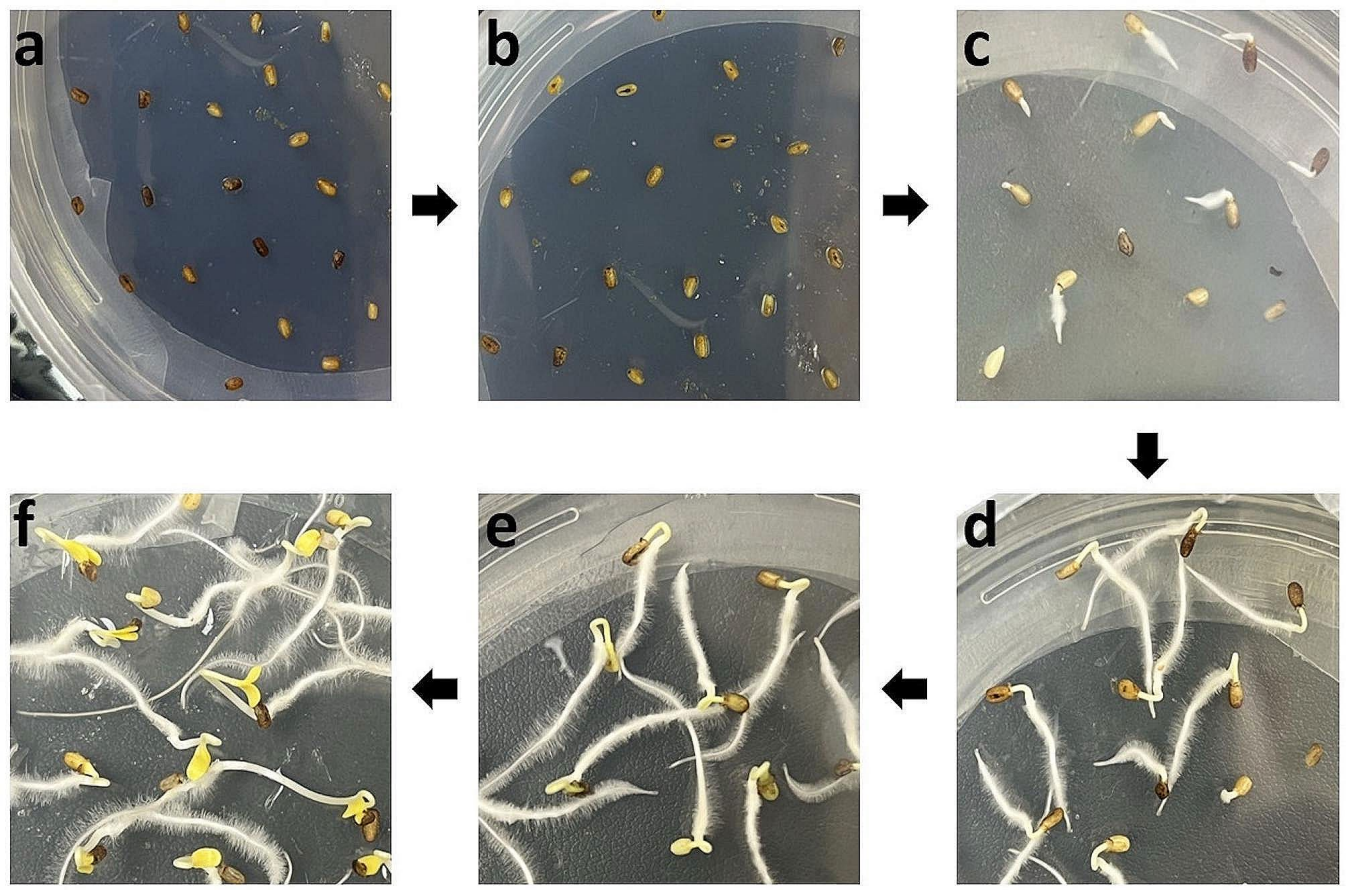
Germination of *C. roseus* (cv. Little Bright Eye) seeds. Phenotype of *C. roseus* seeds/seedlings germinated on half-strength MS medium. **a-f**, the flow of seed germination from 0 (**a**) to 5 days (**f**) of growth

**Fig. 2:**
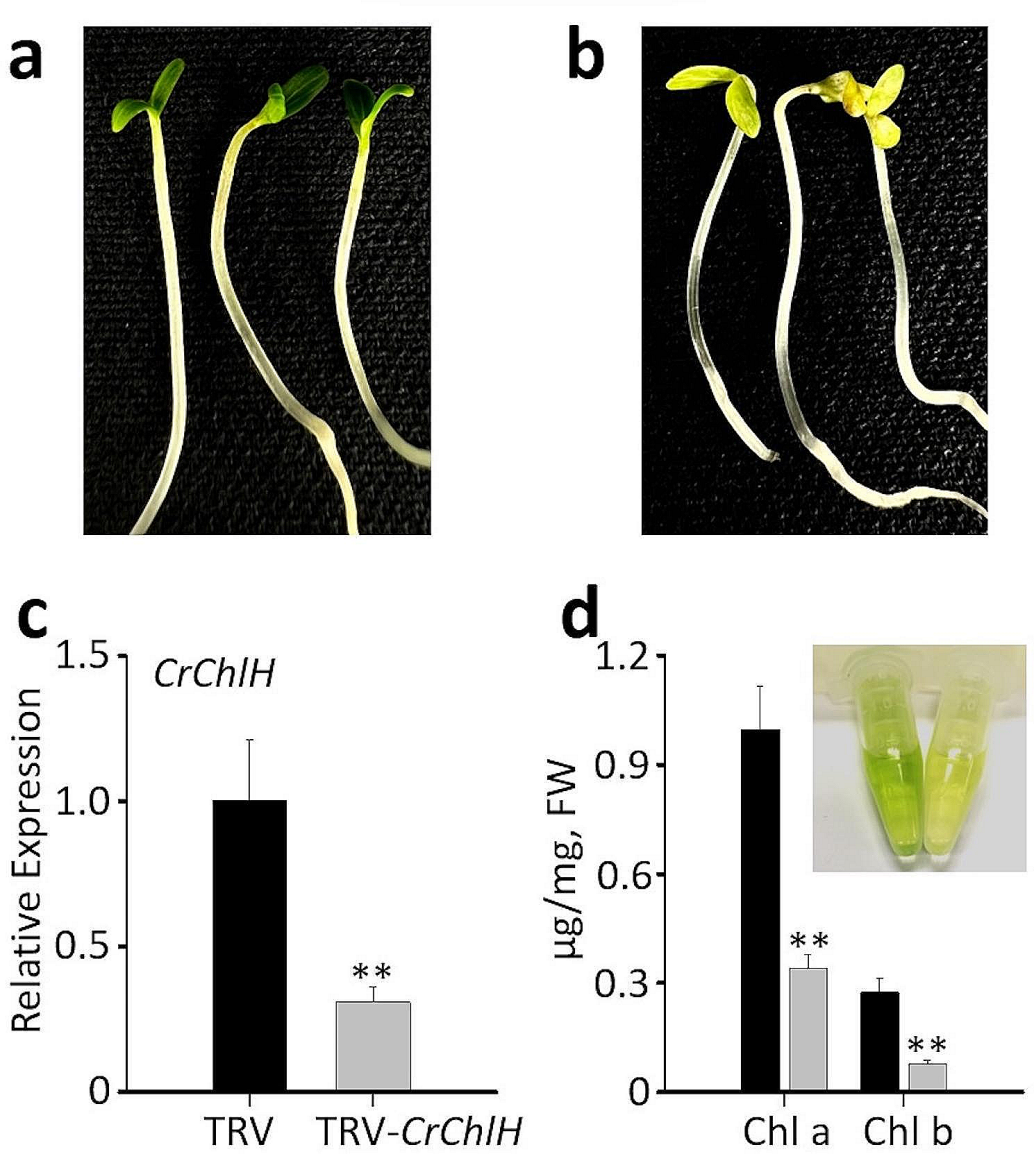
Cotyledon-VIGS of *CrChlH*. Phenotypes of the empty vector (TRV) control (**a**) and *CrChlH*-VIGS (TRV-*CrChlH*) (**b**) seedlings show the yellow cotyledons of *CrChlH*-VIGS seedlings. (**c**) Relative expression of *CrChlH* in control (TRV) and *CrChlH*-VIGS (TRV-*CrChlH*) cotyledons. (**d**) Concentrations of chlorophylls a (Chl a) and b (Chl b) in control (black) and *CrChlH*-VIGS (gray) cotyledons. Inserted picture shows the color difference of the chlorophyll solutions in the control (left) and TRV-*CrChlH* (right). *CrChlH* expression was measured using RT-qPCR. The *C. roseus RPS9* gene was used as an internal reference gene. The values represent means ± SD from three biological replicates. For each biological replicate, entire cotyledons were pooled from 8–9 seedlings (16–17 cotyledons). Statistical significance was calculated using Student’s t test (** *P* < 0.01)

The *C. roseus* seeds were germinated in the dark (Fig. 1a-f). The radicles were emerged from the seed coats on the second day (Fig. 1c), while the cotyledons fully emerged on the fifth day (Fig. 1f). For VIGS, two commonly used marker genes, *protoporphyrin IX magnesium chelatase subunit H* (*CrChlH*), involved in chlorophyll biosynthesis [47], and *phytoene desaturase* (*CrPDS*), a key enzyme in the carotenoid biosynthetic pathway [48], were targeted to generate visible phenotypes. Seedlings or sprouts that were 2, 3, 4, and 5 days old were subjected to vacuum infiltration with *Agrobacterium* (OD_600_ = 1.0) harboring the TRV vectors for a duration of 30 min. Following the infiltration, the sprouts or seedlings were kept in the dark until they were 8-day-old and then exposed to light. A clear *yellow* phenotype was observed in cotyledons after silencing *CrChlH*, when the seedlings were first grown in the dark, followed by 2–3 days of light exposure. The cotyledons of the seedling infiltrated with the *CrChlH*-VIGS construct stayed yellow, whereas that of the control seedlings became green (Fig. 2a-b). Chlorophyll biosynthesis is light-regulated, and as expected, seedlings grown in the dark rapidly accumulate chlorophyll after exposure to light. In our *ChlH* -VIGS study, it was difficult to visually observe the *yellow* phenotype in the light-grown seedlings (2 days of gemination in the dark followed by 3 days in light) even if the *ChlH* expression was significantly reduced (Additional file 1: Fig. S1a and 1b) because of the high chlorophyll content in the cells. Therefore, we carried out *ChlH*-VIGS in dark-grown seedlings initially (5 days in the dark) before exposing the seedlings to light. The decreased expression of *ChlH* prior to the light treatment yielded yellow cotyledons due to reduced chlorophyll accumulation. The *PDS* gene is often used as a marker in VIGS in many plant species including *C. roseus* [12]; however, we did not observe photobleaching in the seedlings infiltrated with *CrPDS*, although *CrPDS* expression was reduced by approximately 70% in the cotyledons (Additional file 1: Fig. S1a and 1b). However, photobleaching was observed in the first pair of true leaves after the seedlings were transferred to soil (Additional file 1: Fig. S1c). The lack of phenotype in the cotyledon is possibly due to the age of the seedlings used in this study. *CrChlH* thus is a more suitable marker for cotyledon VIGS in plant species. The efficiency of silencing *CrChlH* was the highest (84%) when 5-day-old seedlings were used for infiltration (Table 1; Additional file 1: Fig. S2), indicating that complete emergence of the cotyledons from the seed coats is necessary for efficient *Agrobacterium* infection. To optimize the efficiency of cotyledon-VIGS, different OD_600_ values of the *Agrobacterium* infiltration solution were tested. The best result was achieved when the OD_600_ value was at 0.5, resulting in 100% efficiency (Table 1). This optimized condition was then used for subsequent VIGS experiments in *C. roseus*.

**Table 1:** Optimization of cotyledon-VIGS of *CrChlH* in *C. roseus* using different time (days) and varying *Agrobacterium* concentration (OD_600_).

Optimization Parameter (day-OD_600_)	Yellow cotyledon efficiency*
Set 1	
2d-1.0	3/50
3d-1.0	13/50
4d-1.0	24/50
5d-1.0	42/50
Set 2	
5d-0.2	26/50
5d-0.5	50/50
5d-1.0	37/50
5d-2.0	28/50

*The efficiency is shown as the number of yellow cotyledons per 50 cotyledons. Data presented here are from three biological replicates. For each biological replicate, all cotyledons from 9 seedlings (18 cotyledons/replicate) were pooled to determine the phenotype

To confirm the silencing of *CrChlH* through cotyledon-VIGS, *CrChlH* expression in cotyledons was measured using reverse transcription quantitative PCR (RT-qPCR). As expected, the expression of *CrChlH* was reduced by 70% in the *CrChlH*-VIGS cotyledons compared to the control (Fig. 2c). In addition, the contents of chlorophyll a (Chla) and chlorophyll b (Chlb) were decreased in *CrChlH*-VIGS cotyledons (Fig. 2d). These results confirmed that the yellow phenotype of the cotyledons was due to the reduction in chlorophyll contents resulted from silencing *CrChlH*. To further determine the *CrChlH*-VIGS phenotype in the first pair of true leaves and subsequent development, we grew the seedlings in soil. However, only about 20% of the plants showed the *yellow* phenotype in the first pair of true leaves (Additional file 1: Fig. S3a), and the following pair of leaves did not show the phenotype (Additional file 1: Fig. S3b). The results suggest that the virus cannot spread efficiently to the newly emerged leaves in *C. roseus*.

### Cotyledon-VIGS of *CrGATA1* in *C. roseus*

**Fig. 3:**
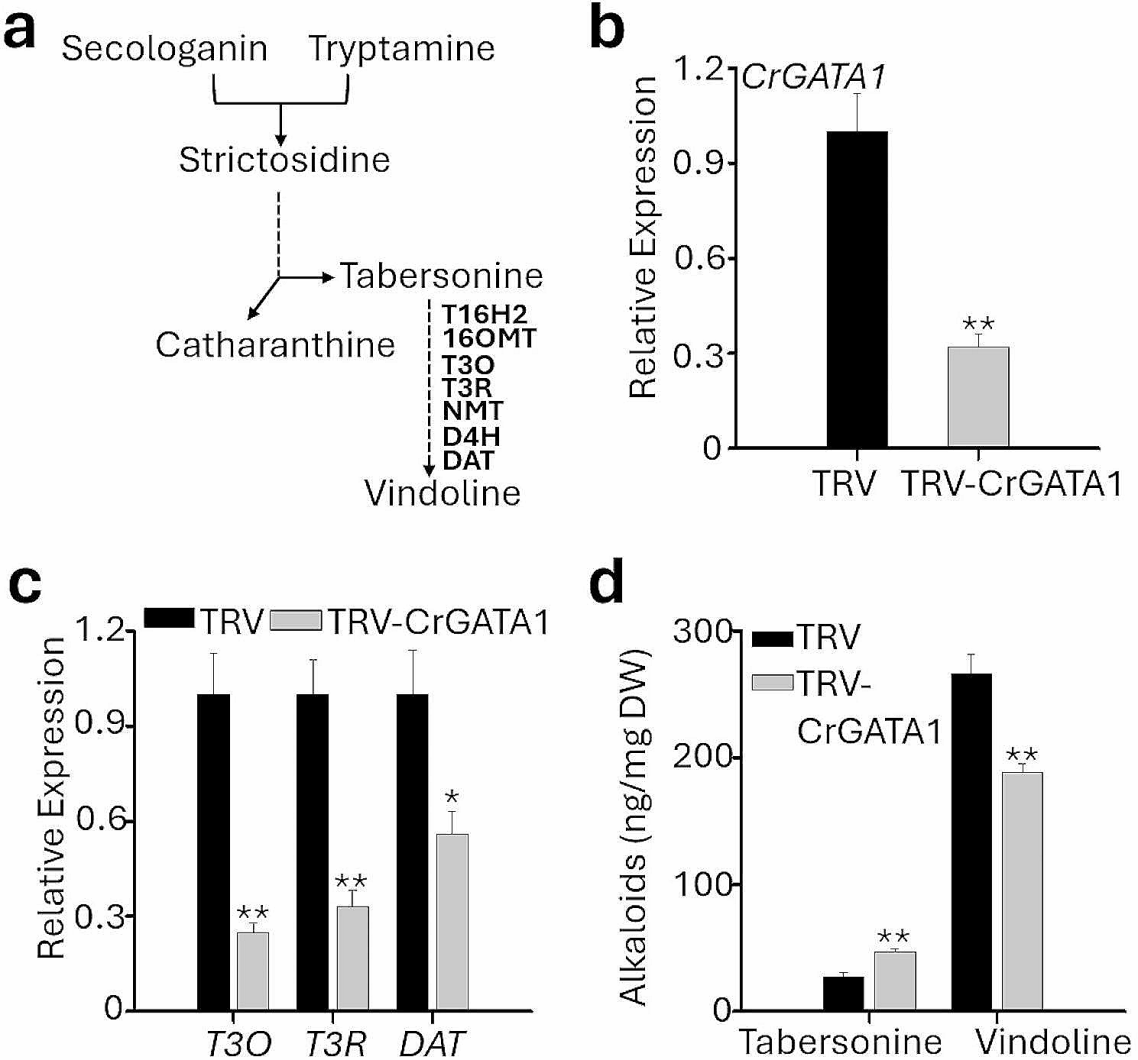
Cotyledon-VIGS of *CrGATA1.* (**a**) A shematic diagram showing vindoline biosynthetic pathway in *C. roseus*. T16H2, tabersonine 16-hydroxylase 2; 16OMT, 16-hydroxytabersonine *O*-methyltransferase; T3O, tabersonine 3-oxygenase; T3R, tabersonine 3-reductase; NMT, 3-hydroxy-16-methoxy-2,3-dihydrotabersonine *N*-methyltransferase; D4H, desacetoxyvindoline-4-hydroxylase; DAT, deacetylvindoline-4-*O*-acetyltransferase. (**b**) Relative expression of *CrGATA1* in empty vector control (TRV) and *CrGATA1*-VIGS (TRV-*CrGATA1*) cotyledons. (**c**) Relative expression of *T3O*, *T3R* and *DAT* in the control and *CrGATA1*-VIGS cotyledons. (**d**) Contents of tabersonine and vindoline in the control and *CrGATA1*-VIGS cotyledons. Gene expression was measured using RT-qPCR, and the *C. roseus RPS9* gene was used as an internal reference gene. Alkaloids were extracted and analyzed by LC-MS/MS, and the concentrations of the alkaloids were estimated based on peak areas compared with standards. DW, dry weight. The values represent means ± SD from three biological replicates. For each biological replicate, entire cotyledons were pooled from 8–9 seedlings (16–17 cotyledons). Statistical significance was calculated using Student’s t test (* *P* < 0.05 and ** *P* < 0.01). The *black* and *grey* bars represent the TRV (empty vector control) and TRV-*CrGATA1*, respectively

The sequential conversion of tabersonine to vindoline is catalyzed by seven genes encoding enzymes tabersonine 16-hydroxylase 2 (T16H2), 16-hydroxytabersonine *O*-methyltransferase (16OMT), tabersonine 3-oxygenase (T3O), tabersonine 3-reductase (T3R), 3-hydroxy-16-methoxy-2,3-dihydrotabersonine *N*-methyltransferase (NMT), desacetoxyvindoline-4-hydroxylase (D4H), and deacetylvindoline-4-*O*-acetyltransferase (DAT) [49] (Fig. 3a). In our previous study, we have demonstrated that the expression of *CrGATA1*, a positive regulator of vindoline biosynthesis, can be effectively knocked down using the pinch wounding VIGS method. VIGS of *CrGATA1* reduced the expression of *T3O*, *T3R*, and *DAT* [13]. In this study, we further validated the applicability of cotyledon-VIGS in *C. roseus* by targeting *CrGATA1*. Five-day-old *C. roseus* seedlings (germinated in dark for 2 days followed by 3 days of light) were vacuum-infiltrated and then incubated in dark for 3 days and in light for another 3 days. The conditions for growing *C. roseus* seedlings used for silencing TIA related genes were different from those used for *CrChlH*-VIGS. This is because TIA biosynthesis (especially vindoline) requires light (darkness inhibits vindoline production). The expression of *CrGATA1* reduced by approximately 70% in *CrGATA1*-VIGS cotyledons (Fig. 3b). Consistent with our previous findings [13], the expression of the vindoline pathway genes, *T3O*, *T3R*, and *DAT*, was significantly downregulated in the *CrGATA1*-VIGS cotyledons (Fig. 3c). Furthermore, we detected a decrease of vindoline and an increase of tabersonine, the precursor of vindoline synthesis, in the *CrGATA1*-VIGS cotyledons (Fig. 3d), which is in agreement with our previous results using the pinch wounding VIGS method [13]. These results further validate the application of cotyledon-VIGS in *C. roseus* for functional characterization of the pathway genes.

### Cotyledon-VIGS of *CrMYC2* combined with MeJA treatment

**Fig. 4:**
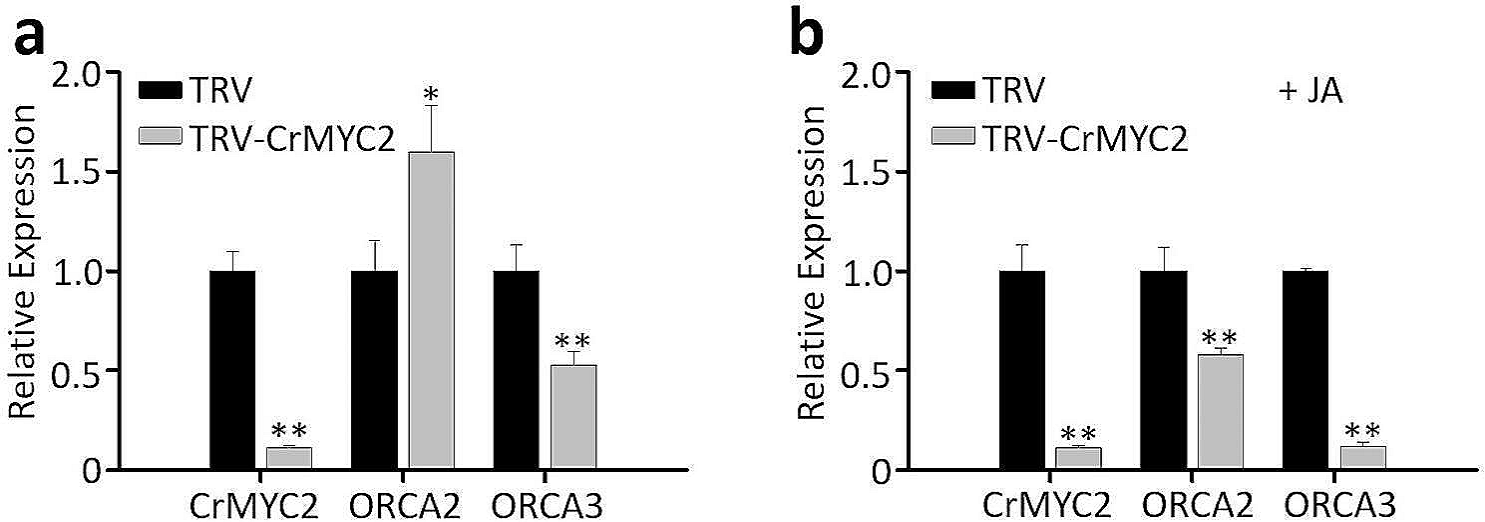
Cotyledon-VIGS of *CrMYC2* with or without MeJA treatment. Relative expression of *CrMYC2*, *ORCA2*, and *ORCA3* in empty vector control (TRV) and *CrMYC2*-VIGS (TRV-*CrMYC2*) cotyledons without (**a**) or with (**b**) MeJA treatment (+ MeJA; 100 µM). MeJA (100 µM) was added to the petri dishes containing the seedlings and petri dishes covered with the lids for 2 h. Gene expression was measured using RT-qPCR. The *C. roseus RPS9* gene was used as an internal reference gene. The values represent means ± SD from three biological replicates. For each biological replicate, entire cotyledons were pooled from 8–9 seedlings (16–17 cotyledons). Statistical significance was calculated using Student’s t test (** *P* < 0.01)

CrMYC2 is a component of jasmonate signaling and a key regulator of the TIA pathway. In our cotyledon-VIGS experiments, expression of *CrMYC2* was knocked down by 90% (Fig. 4a). However, only *ORCA3* showed a 40% reduction in expression, whereas *ORCA2* expression was higher in *CrMYC2*-VIGS cotyledons compared to the control (Fig. 4a). Subsequently, we treated the *CrMYC2*-VIGS cotyledons with 100 µM MeJA for 2 h before collecting samples. The results showed that both *ORCA2* and *ORCA3* were significantly downregulated upon *CrMYC2* silencing, with *ORCA3* showing an 80% reduction compared to the 40% reduction without MeJA treatment (Fig. 4b). In *C. roseus*, the expression of *CrMYC2*, *ORCA2*, and *ORCA3* is induced by MeJA, and CrMYC2 is essential for MeJA-responsive expression of *ORCAs* [34]. In addition, other factors, such as AT-hook proteins, are known to regulate *ORCA* expression [50]. In the absence of MeJA, minimum expression of *CrMYC2* in VIGS seedlings had little to no significant effect on the expression of *ORCA2* and *ORCA3*. Our results agreed with the previously published findings [34] showing that without MeJA treatment, RNAi-mediated silencing of *CrMYC2* in *C. roseus* cell lines has no significant effect on the expression of *ORCA2* and *ORCA3;* however, MeJA treatment significantly affected the expression of *ORCA2* and *ORCA3* compared to control. Additionally, our findings suggest that when conducting cotyledon-VIGS in *C. roseus*, the seedlings are amenable to other treatments, such as other phytohormones or stress conditions, providing opportunities for further investigations.

### Simultaneous VIGS of *CrGBF1/2* and overexpression of *CrMYC2*

**Fig. 5:**
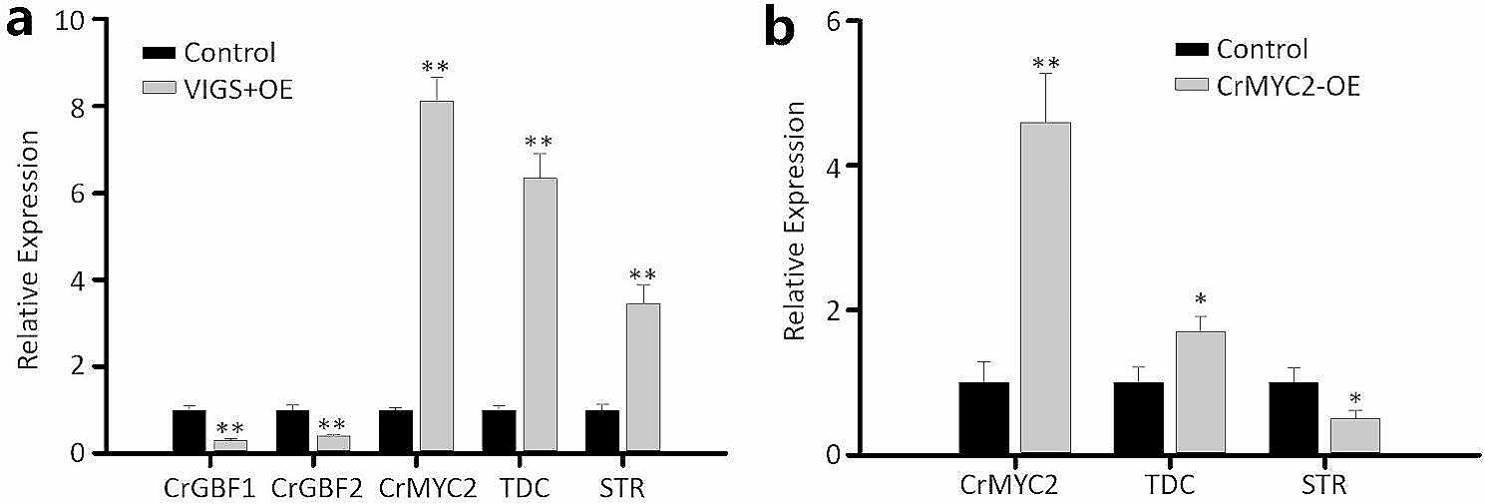
Cotyledon-VIGS of *CrGBF1/GBF2* and overexpression of *CrMYC2.* (**a**) Relative expression of *CrGBF1, CrGBF2*, *CrMYC2*, *TDC* and *STR* in empty vector control (TRV) and *CrGBF1/GBF2/MYC2* cotyledons. (**b**) Relative expression of *CrMYC2*, *TDC* and *STR* in empty vector control and *CrMYC2* overexpression cotyledons. Relative expression was measured by RT-qPCR, and the *C. roseus RPS9* gene was used as an internal reference gene. The values represent means ± SD from three biological replicates. For each biological replicate, entire cotyledons were pooled from 8–9 seedlings (16–17 cotyledons). Statistical significance was calculated using Student’s t test (** *P* < 0.01)

Previous studies have established a seedling-based transient overexpression method for *C. roseus* using vacuum infiltration [51]. Here, we aimed to explore the possibility of simultaneously achieving gene silencing and transient gene overexpression in *C. roseus* seedlings. CrGBF1/2 are negative regulators of TIAs biosynthesis, and CrMYC2 works antagonistically with CrGBF1/2 to regulate TIAs biosynthesis [35]. We hypothesized that overexpression of *CrMYC2* while silencing *CrGBF1/2* would maximize the levels of TIA biosynthesis. For cotyledon-VIGS, gene fragments of *CrGBF1* and *CrGBF2* were fused to achieve simultaneous silencing of both genes. The *Agrobacterium* solutions for *GBF1/2*-VIGS and *CrMYC2*-overexpression (OE) were mixed in an equal proportion prior to vacuum infiltration. Five-day-old seedlings (germinated in dark for 2 days then kept in light for 3 days) were used for *Agrobacterium*-infiltration, and the resulting seedlings were kept in the dark for 3 days and then in light for another 3 days before measuring gene expression. Our results showed that *CrMYC2* was overexpressed by 8-fold, while CrGBF1/2 were knocked down by 60–70% in VIGS + OE seedlings (Fig. 5a). Tryptophan decarboxylase (TDC) and strictosidine synthase (STR), two enzymes in the TIA pathway, are the targets of CrMYC2 and CrGBF1/2. Tryptophan is decarboxylated by *TDC* to produce tryptamine, the indole moiety of TIA. Condensation of tryptamine with the terpenoid moiety secologanin to produce the first TIA, strictosidine, is catalyzed by *STR* [52]. The expression of *TDC* and *STR* was induced significantly (4–6 fold) in the VIGS + OE cotyledons (Fig. 5a). However, *TDC* expression was induced moderately (2-fold) whereas that of *STR* was repressed when only CrMYC2 was overexpressed (Fig. 5b). The expression of *MYC2* increased 8-fold in VIGS + OE seedlings compared to control whereas it increased 5-fold in *MYC2*-OE seedlings (Fig. 5a and b). This difference in *MYC2* expression could possibly be the effect of silencing of the GBFs in VIGS + OE seedlings. These findings suggest that the cotyledon-VIGS method can be combined with transient gene overexpression to achieve simultaneous up- and down-regulation of desired genes in *C. roseus*.

### Application of cotyledon-VIGS in *G. inflata* and *A. annua*

**Fig. 6:**
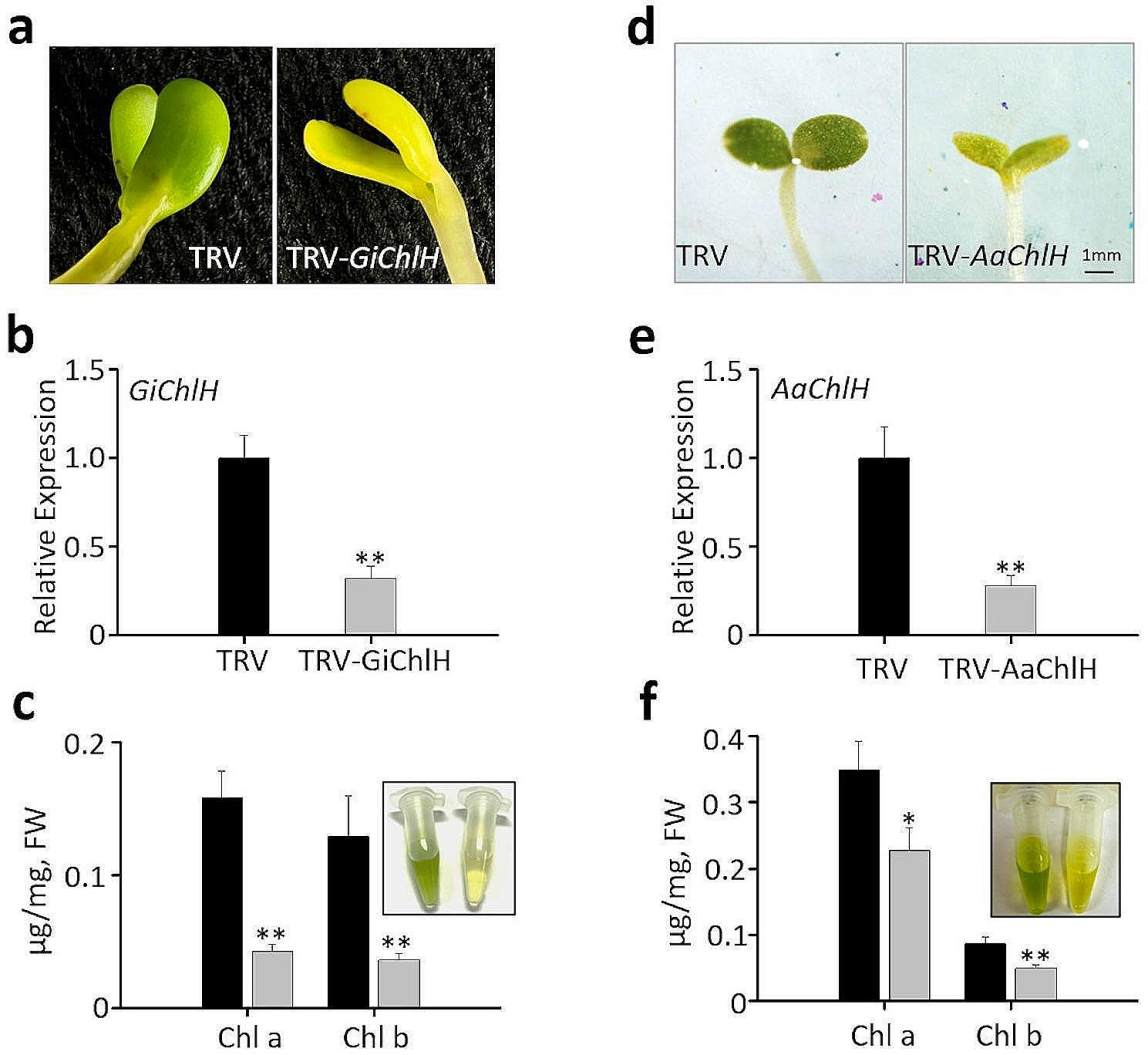
Cotyledon-VIGS of *ChlH* genes in *G. inflata* and *A. annua*. (**a**) Phenotypes of empty vector control (TRV) and *GiChlH*-VIGS (TRV-*GiChlH*) seedlings. (**b**) Relative expression of *GiChlH* in the control and *GiChlH*-VIGS cotyledons. (**c**) Concentration of chlorophylls a (Chl a) and b (Chl b) in the control and *GiChlH*-VIGS cotyledons. (**d**) Phenotypes of TRV control and *AaChlH*-VIGS (TRV-*AaChlH*) seedlings. (**e**) Relative expression of *AaChlH* in the control and *AaChlH*-VIGS cotyledons. (**f**) Concentration of Chl a and Chl b in control and *AaChlH*-VIGS cotyledons. In **a** and **d**, the yellow cotyledons of the VIGS seedlings are consistent with the chlorophyll extractions showing in the inserts of **c** and **f** (Left, TRV control; right, *ChlH*-VIGS). Relative expression was measured using RT-qPCR, and the *A. annua* and *G. inflata Actin* genes were used as an internal reference. The values represent means ± SD from three biological replicates. For each biological replicate, entire cotyledons were pooled from 8–9 seedlings (16–17 cotyledons) for *A. annua*, and 4–5 seedlings (8–10 cotyledons) for *G. inflata*. Statistical significance was calculated using Student’s t test (* *P* < 0.05 and ** *P* < 0.01)

The success of cotyledon-VIGS in *C. roseus* prompted us to apply this method to other plants including *G. inflata* and *A. annua*, two important medicinal plants. We initially used the conditions that worked well for *C. roseus* (30 min infiltration with OD_600_ 0.5 *Agrobacterium* solution). However, silencing efficiency was low for *G. inflata* possibly because the cotyledons are very thick. Therefore, for *G. inflata*, the infiltration time was increased to 60 min and the concentration of infiltration solution was increased to OD_600_ = 1.0 to achieve the best efficiency (Table 2). In contrast, *A. annua* seedlings are sensitive to long exposure (i.e. 30 min) to *Agrobacterium* infiltration. We thus reduced the infiltration time to 10 min for *A. annua* (Table 2). Six to seven-day-old seedlings germinated in dark were used for VIGS. The respective *ChlH* genes were used for cotyledon-VIGS in both plants, and we observed yellow-colored cotyledons with 100% efficiency (Table 2; Fig. 6a and d), which was confirmed by measuring the chlorophyll concentration and gene expression (Fig. 6b, c, e and f). Based on these results, we conclude that cotyledon-VIGS is a promising and generally applicable technique for investigating gene function in plants.

**Table 2:** Optimized conditions of the cotyledon-VIGS in two medicinal plants

Plants/Conditions	Glycyrrhiza inflata	Artemisia annua
Seedling age (days)	7	6
Infiltration time (minutes)	60	10
OD_600_ of infiltration solution	1.0	0.5
Efficiency*	30/30	50/50

*After infiltration with the VIGS vectors, the seedlings were incubated for 6 days (3 days in dark and then 3 days in 16 h light /8 h dark regime), to record yellow cotyledon phenotype. The efficiency is shown as the number of yellow cotyledons per 30–50 cotyledons

## Discussion

VIGS is a valuable tool for plant functional genomics and has been extensively used to decipher the gene functions in developmental and metabolic pathways [1–3, 12, 13, 15]. VIGS is especially useful for non-model plant species for which the generation of stable transgenic plants is often challenging [1]. To enhance the applicability of VIGS, various infiltration methods have been developed, among which the sprout vacuum infiltration (SVI) method allows for high-throughput gene function analysis [25]. SVI-based VIGS and other seed-based infiltration methods [10, 18] are rapid and the bleaching phenotype is usually easy to observe in the first pair of true leaves. However, for plants with a prolonged developmental period, the application of SVI method is more time consuming. For *C. roseus*, the first pair of true leaves appear 3 weeks after germination [51]. The cotyledon-VIGS method (Fig. 2) circumvents this issue and maximizes the efficiency of the VIGS. In our laboratory, 5-day-old *C. roseus* seedlings were used for *Agrobacterium*-infiltration and samples were ready for collection 6 days after infiltration. Moreover, cotyledon-VIGS retains the high-throughput advantage of SVI. The efficiency of VIGS varies for each individual gene. The silencing efficiency of genes varies from 60 to 90% in our study (Figs. 3, 4, 5 and 6).

Previous studies have reported varying efficiency of different infiltration methods in different plant species. For instance, while SVI has been highly effective in certain *Solanaceous* crops, such as tomato and eggplant [25], its efficiency in two *Goji* species (*Lycium* species), which also belong to *Solanaceae*, is notably lower [28]. The pinch wounding method has been found to be suitable for *C. roseus* [11]; however, its efficiency is difficult to determine, leading to the development of an improved method in which the marker gene *CrPDS* is simultaneously silenced with the target gene to visualize the gene silencing effect [16]. The inconsistency in silencing may be attributed to the requirement of the virus to spread through vascular tissue to the distant plant tissues. We found that the VIGS efficiency greatly declined in the newly emerged leaves (Additional file 1: Fig. S3). In contrast, cotyledon-VIGS does not necessitate long-distance viral spread, making it highly efficient in diverse plant species. We demonstrated 100% efficiency in cotyledon-VIGS for *C. roseus*, *G. inflata*, and *A. annua* (Tables 1 and 2), suggesting its potential as a general and efficient VIGS method for most plant species.

*C. roseus* accumulates two valuable anti-cancer agents, vinblastine and vincristine, specifically in leaves, with catharanthine and vindoline being their direct precursors. Understanding the regulatory mechanisms underlying the biosynthesis of catharanthine and vindoline can serve as a foundation for improving vinblastine and vincristine production. Although vinblastine and vincristine are not accumulated in the cotyledon of *C. roseus*, catharanthine and vindoline are readily produced [53]. Cotyledon-VIGS of *CrGATA1* reiterated the positive effects of CrGATA1 in regulating vindoline biosynthesis (Fig. 3). Additionally, CrMYC2 and its targets, ORCAs, act as general regulators of catharanthine and most tryptamine-derived indole alkaloids upstream of vindoline [34]. Cotyledon-VIGS of *CrMYC2*, followed by JA treatment, further validated the effects of CrMYC2 on its target genes and the involvement of the JA signaling (Fig. 4). Therefore, cotyledon-VIGS provides a platform for investigating the regulatory mechanisms of catharanthine and vindoline, as well as other upstream TIAs.

The combination of cotyledon-VIGS with transient overexpression in *C. roseus* seedlings allows for investigating the relationship between multiple factors in a pathway, even in non-model plants where stable transformation is challenging. By overexpressing the activator *CrMYC2* and simultaneously knocking down two repressors, *CrGBF1* and *CrGBF2*, using cotyledon-VIGS, we observed a greater upregulation of *TDC* and *STR* compared to control and the individual gene manipulation (Fig. 5).

Cotyledon-VIGS overcomes several issues facing the previously established VIGS methods and can be used for other non-model plant species. Although a protocol optimized for one species might work for other species, the parameters still need to be fine-tuned for each plant species to achieve optimal results. Each plant species is different with respect to germination time, size, and morphology of the cotyledon, as well as the sensitivity to *Agrobacterium* infection. In our study, the parameters that worked well for *C. roseus* did not yield the best results for *G. inflata* and *A. annua*. Therefore, certain conditions, such as age of the seedling, density (OD_600_) of *Agrobacterium*-suspension, and infiltration time, should be optimized for each plant species to achieve optimal results. It is reasonable to suggest cotyledon-VIGS as a general platform for gene silencing and investigation of the synergistic effects of multiple genes. Cotyledon-VIGS is most effective in studying genes that are expressed in early development. Nonetheless, we were also able to obtain cotyledon-VIGS *C. roseus* plants that developed true leaves, making the system potentially useful for studying late stage-expressed genes.

## Methods

### Plant materials and growth conditions

Seeds of *C. roseus* (cultivar ‘Little Bright Eye’; obtained from NESeed, USA) were used in this study. The seeds were surface sterilized using 75% ethanol for 5 min and then 30% sodium hypochlorite solution (Sigma-Aldrich) for 10 min. After rinsing with sterile ddH_2_O for 5 times, the seeds were inoculated on half-strength Murashige and Skoog (½ MS) medium. The seeds were kept in the dark at 30 °C for two days and then transferred to an incubator at 26 °C. For VIGS experiments targeting the *CrChlH* (accession numbers HQ608936) and *CrPDS* (accession number JQ655739) in *C. roseus*, the germinated seeds were grown in the dark for another 3 days. However, for VIGS of TIA pathway genes, the germinated seeds were grown under a light regime of 16/8 photoperiod for 3 days.

For VIGS of *ChlH* genes in *G. inflata*, and *A. annua*, the seeds of respective species were germinated on half-strength MS medium, and seedlings were grown in the dark at 26 ℃. Seeds of *G. inflata* were treated with H_2_SO_4_ for 30 min [46], surface sterilized with 30% sodium hypochlorite solution (Sigma-Aldrich) for 10 min, and germinated on half-strength MS medium for 7 days in dark. Seeds of *A. annua* were surface sterilized as described for *C. roseus* seeds and germinated on half-strength MS medium for 6 days in dark.

### Plasmid construction and *Agrobacterium* transformation

The primers used for plasmid construction are listed in Additional file 1: Table S1 and the vectors are schematically presented in Additional file 1: Figure S4. For VIGS vectors, fragments of target genes were amplified with primers containing KpnI and XhoI restriction enzyme recognition sites and inserted into the multiple cloning sites (MCS) of pTRV2 [9]. Fragments of *CrGBF1* (accession numbers AF084971) and *CrGBF2* (accession numbers AF084972) were fused together using primers with overlapping sequences. The VIGS vectors for silencing *CrChlH*, *CrPDS* [12], *CrGATA1* [13], and overexpressing *CrMYC2* (accession number AF283507) [35] have been described previously. The *ChlH* gene sequences of *G. inflata* and *A. annua* were obtained from an unpublished *G. inflata* transcriptome and a published *A. annua* transcriptome [54], respectively (Additional file 1: Supplementary text).

*Agrobacterium tumefaciens* strain GV3101 competent cells stored at -80 °C were thawed on ice and then mixed with 500 ng of recombined plasmids. The mixture was kept on ice for 30 min and then rapidly frozen in liquid nitrogen for 30 s, followed by incubation at 37 °C for 5 min. The cells were returned to ice for 5 min and quenched with 500 µL of fresh Luria Broth (LB) liquid medium. Following incubation in a shaker at 28 °C and 200 rpm for 2.5 h, 100 µL of cells were plated onto LB agar plates containing rifampicin (30 mg/L) and kanamycin (100 mg/L) and incubated at 28 °C for 3 days.

### *Agrobacterium* culture and preparation of infiltration

A single positive colony of transformed *Agrobacterium* was inoculated into 1 mL of LB liquid medium containing 30 mg/L rifampicin and 100 mg/L kanamycin, followed by overnight culturing in a shaker at 28 °C with a speed of 200 rpm. Subsequently, 100 µL of *Agrobacterium* cells were transferred into 10 mL of fresh LB liquid medium supplemented with the aforementioned antibiotics and cultured overnight at 28 °C with a speed of 200 rpm. The *Agrobacterium* cultures were then centrifuged at 6000 g for 5 min, and the resulting pellet was resuspended in an infiltration buffer containing 10 mM MgCl_2_, 10 mM MES, and 100 µM acetosyringone, at a desired OD_600_ (optical density at 600 nm). The suspension was then incubated at 28 °C for at least 3 h. Afterward, the infiltration solution was mixed with Silwet L-77 at a concentration of 0.01% `and was ready for infiltration. For simultaneous VIGS + OE (overexpression), *Agrobacterium* harboring the *CrGBF*-VIGS and *CrMYC2*-OE constructs were mixed in equal proportions before infiltration.

### Infiltration of the seedlings

Sprouts or seedlings of *C. roseus*, *G. inflata*, and *A. annua* were immersed in the infiltration solution in either a 15 mL or 50 mL tube. The opening of the tube was covered with parafilm that was punctured to produce small holes to allow for air exchange. For VIGS of the *ChlH* or *PDS* gene, the tubes were wrapped with aluminum foil to prevent light exposure (Additional file 1: Figure S5a) and placed in a vacuum chamber. The infiltration was carried out at the desired pressure of 20 kPa and for the appropriate duration (Additional file 1: Figure S5b). Afterward, the pressure was slowly released. Sprouts or seedlings were gently taken out from the tubes, washed with sterile distilled water for five times, and placed on petri dishes with autoclaved wet filter papers. The seedlings were then kept in the dark at 26 °C for 3 days, followed by transferring to light (15–20 µmol m^− 2^ s^− 1^; photoperiod 16/8) for 3 days. For VIGS of the *ChlH* gene, cotyledons were harvested for chlorophyll content determination and RNA isolation, or the seedlings were transferred to soil for further observation. For VIGS of *CrMYC2*, infiltrated *C. roseus* seedlings were treated with 100 µM MeJA for 2 h 6 days after infiltration. For VIGS of *CrGATA1*, infiltrated *C. roseus* seedlings were kept in the dark for 3 days and then in 16 h light/8 h dark for another 3 days. The cotyledons were then collected for gene expression and metabolite analysis.

### Determination of chlorophyll contents

The protocol for chlorophyll content determination has been previously described [55]. Briefly, samples were weighed and placed in 1 mL of dimethyl-formamide (DMF) and kept in the dark at 4 °C overnight. Optical density at 664 nm and 647 nm (A_664_ and A_647_) was measured using a spectrophotometer, using pure DMF as a blank. The contents of chlorophyll a (C_a_) and chlorophyll b (C_b_) were calculated using the following formulas: C_a_ = 11.65×A_664_ – 2.69×A_647_; C_b_ = 20.81×A_647_ – 4.53×A_664_.

### RNA isolation, cDNA synthesis, and RT-qPCR

Total RNA was extracted from *C. roseus* VIGS cotyledons using the RNeasy Plant Mini Kit according to the manufacturer’s instructions (QIAGEN, United States). Approximately 2 µg of total RNA was treated with DNase I to remove contaminating genomic DNA. First-strand cDNA synthesis was carried out using Superscript III reverse transcriptase (Invitrogen, United States) in a total reaction volume of 20 µL. Reverse transcription quantitative PCR (RT-qPCR) was performed to measure the transcript levels of target genes. *CrRPS9* was used as an internal control for normalization [40]. *AaActin* and *GiActin* were used as internal control for *A. annua* and *G. inflata*, respectively. Relative gene expression was determined as previously described [13]. All RT-qPCRs were performed in triplicate and repeated twice to ensure accuracy and reproducibility. The primer sequences used for RT-qPCR are provided in Additional file 1: Table S1.

### Alkaloid extraction and analysis

Extraction and analysis of alkaloids from *C. roseus* VIGS cotyledons were performed as described previously [13]. The concentrations of the alkaloids were calculated using a standard curve.

### Electronic supplementary material

Below is the link to the electronic supplementary material.

Supplementary Material 1

## Data Availability

All data generated or analyzed during this study are included in this published article (and its supplementary information files).
