# Supplementary material for: A Cotyledon-based Virus-Induced Gene Silencing (Cotyledon-VIGS) approach to study specialized metabolism in medicinal plants
Source: Plant Methods. 2024 Feb 12;20:26. doi: 10.1186/s13007-024-01154-x (PMC10860238; doi:10.1186/s13007-024-01154-x)
Supplement: Supplementary file 1 — Supplementary Material 1 [file 13007_2024_1154_MOESM1_ESM.docx]

**Additional File 1**

**A Cotyledon-based Virus-induced Gene Silencing (Cotyledon-VIGS) Approach to Study Specialized Metabolism in Medicinal Plants**

Yongliang Liu, Ruiqing Lyu, Jushua J. Singleton, Barunava Patra, Sitakanta Pattanaik*, Ling Yuan*

Department of Plant and Soil Sciences and Kentucky Tobacco Research and Development Center, University of Kentucky, Lexington, Kentucky 40546

* Ling Yuan and Sitakanta Pattanaik

Email: [lyuan3@uky.edu](mailto:lyuan3@uky.edu); [spatt2@uky.edu](mailto:spatt2@uky.edu)

**a**

**
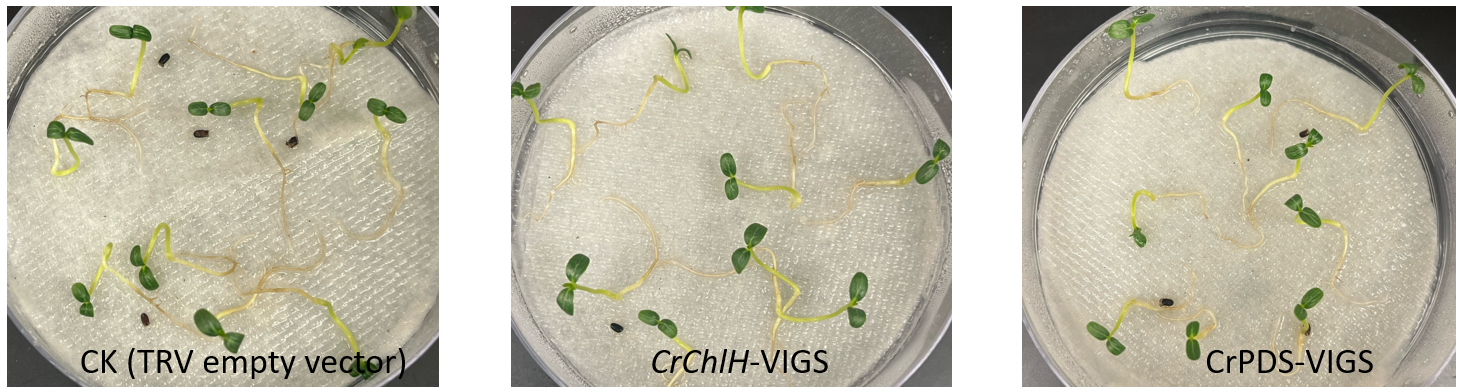
**

**b c**

**
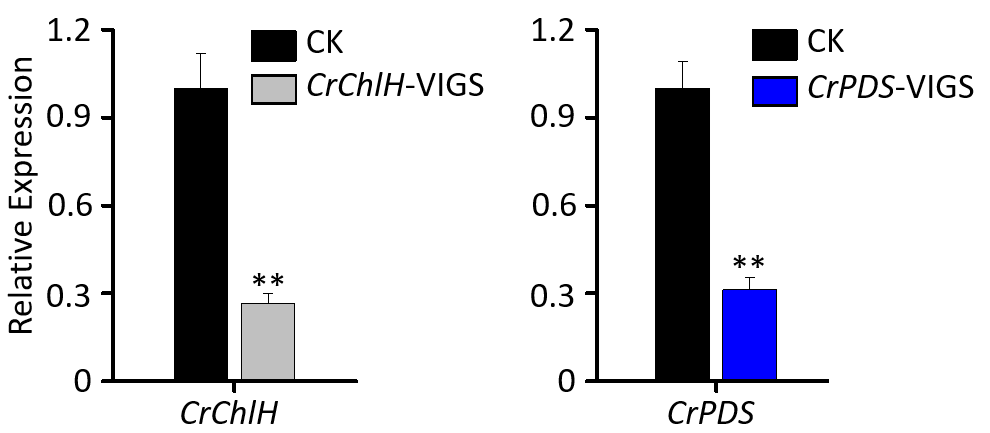

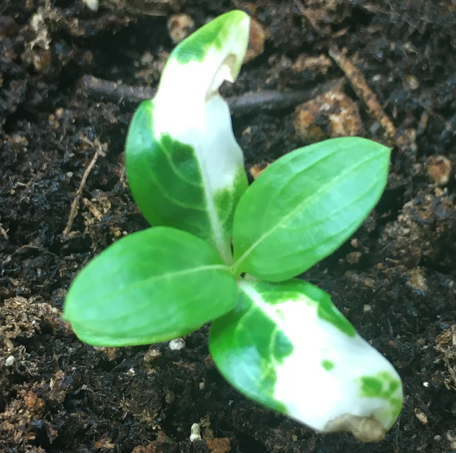
**

**Figure S1** Analyses of phenotypes and gene expression of *CrChlH* or *CrPDS*-VIGS seedlings. **a** Phenotypes of control and *CrChlH* or *CrPDS*-VIGS seedlings grown under normal light condition. For *CrChlH* or *CrPDS*-VIGS, 5-day-old seedlings (germinated in dark for 2 days and then exposed to light for 3 days) were used for infiltration. Following infiltration, seedlings were kept in the dark for 3 days and then changed to a 16h light/8h dark regime for 3 days. Cotyledons were collected from 11-day-old seedlings for gene expression analysis. **b** Relative expression of *CrChlH* and *CrPDS* in control (TRV), *CrChlH*-VIGS and *CrPDS*-VIGS cotyledons collected from 11-day-old seedlings. *CrChlH* and *CrPDS* expression was measured using RT-qPCR. The values represent means ± SD from three biological replicates. Statistical significance was calculated using Student’s t test (** P < 0.01). **c** Phenotype of cotyledon VIGS seedling after transfer to soil. Photo-bleaching phenotype was observed in first pair of true leaves but disappeared in the second pair of leaves.


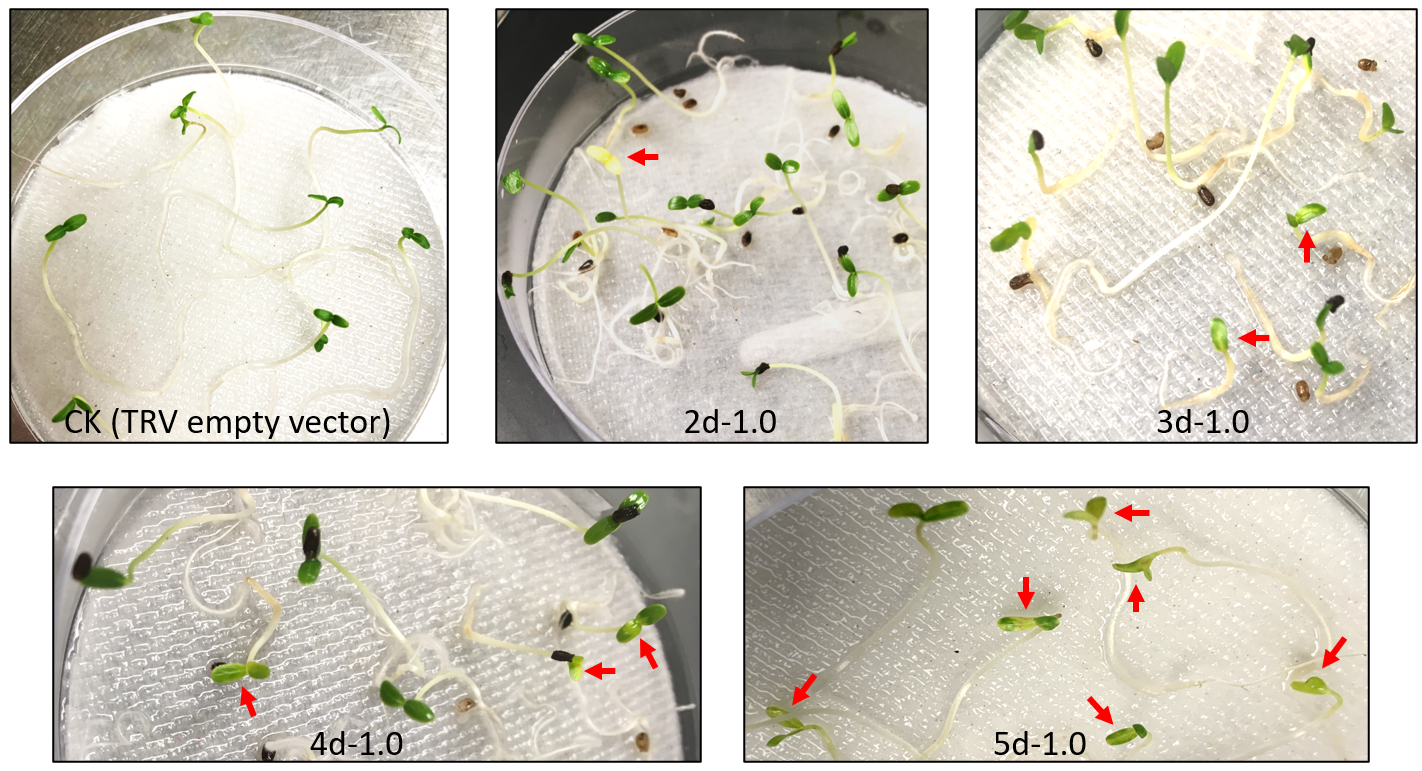


**Figure S2** Variable efficiency of *CrChlH*-VIGS as observed in 2/3/4/5-day-old *C. roseus* seedlings. The *yellow* cotyledon phenotype is significantly higher in 5-day-old seedlings (indicated by *red* arrowheads). Pictures of one representative experiment is shown here.

**
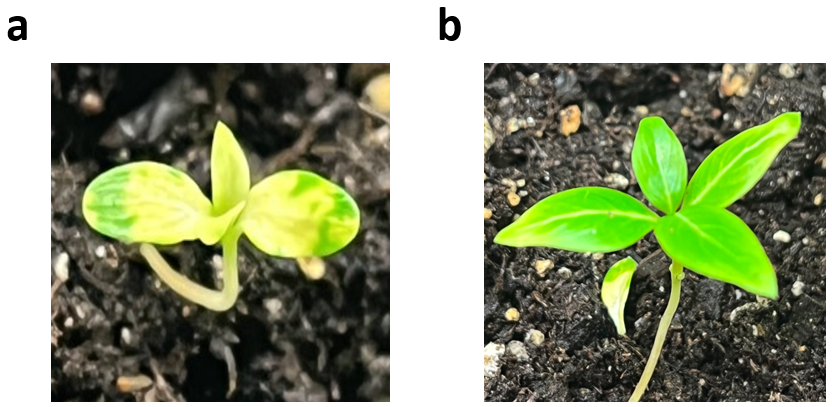
**

**Figure S3** Phenotypes of *CrChlH*-VIGS at true leaf stage. **a** The first pair of true leaves show *yellow* color phenotype like the cotyledons. **b** In the older seedling, the yellow phenotype in the first pair of true leaves mostly disappeared and the second pair of true leaves did not show no yellow phenotype.

**
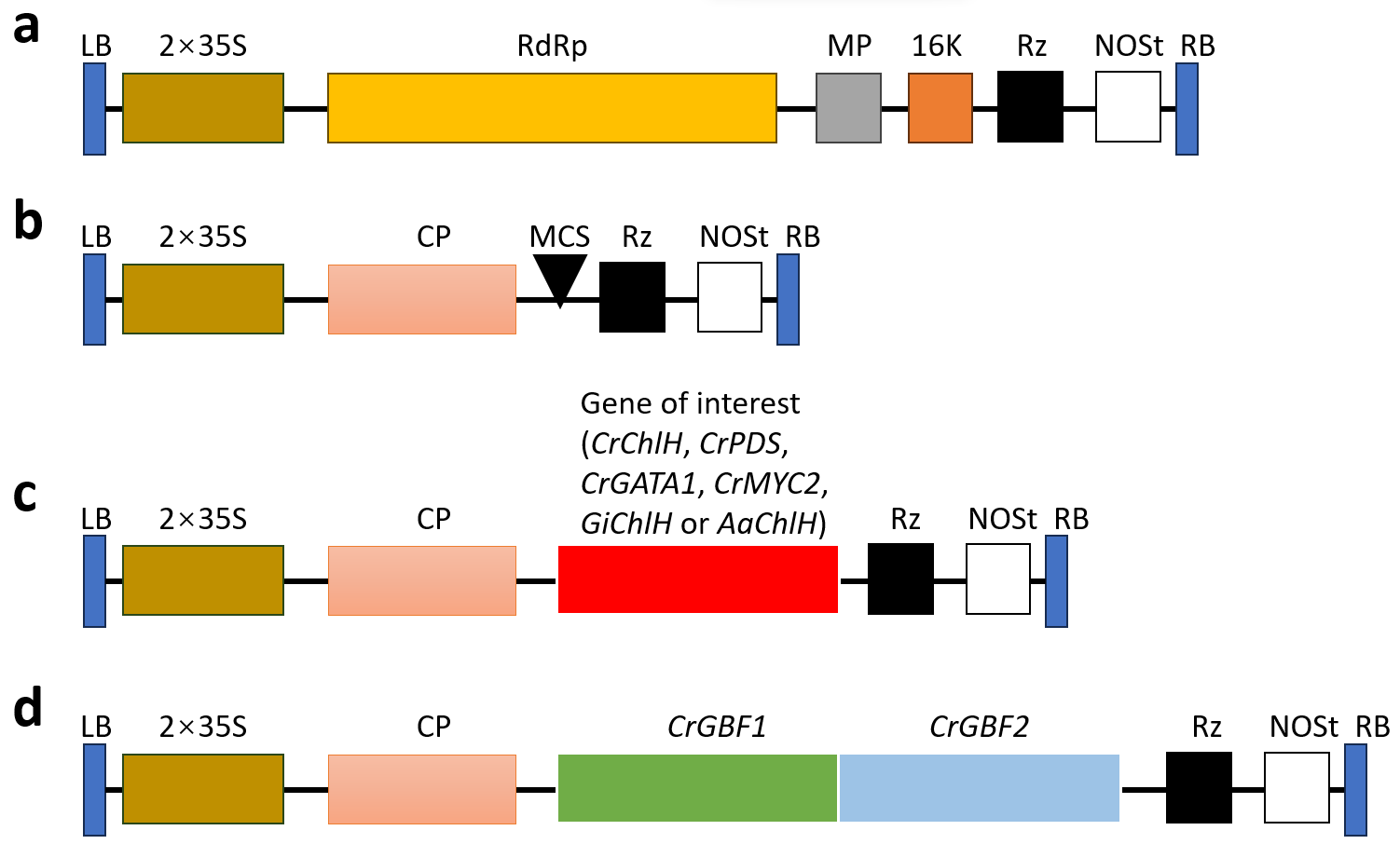
**

**Figure S4** Tobacco rattle virus (TRV)-based virus-induced gene silencing (VIGS) constructs used in this study. **a and b** TRV-based VIGS vector systems. 2×35S, duplicated *CaMV* 35S promoter; NOSt; nopaline synthase terminator; RdRp, RNA-dependent RNA polymerase; 16K, 16 kDa cysteine-rich protein; MP, movement protein; CP, coat protein; LB and RB, left and right borders of T-DNA; Rz, self-cleaving ribozyme; MCS, multiple cloning sites. **c** Map of the TRV2 constructs with the fragment of the gene-of-interest (*CrChlH*, *CrPDS*, *CrGATA1*, *CrMYC2*, *GiChlH*, or *AaChlH*) inserted in the MCS. **d** Map of the pTRV2-CrGBF1/2 construct.


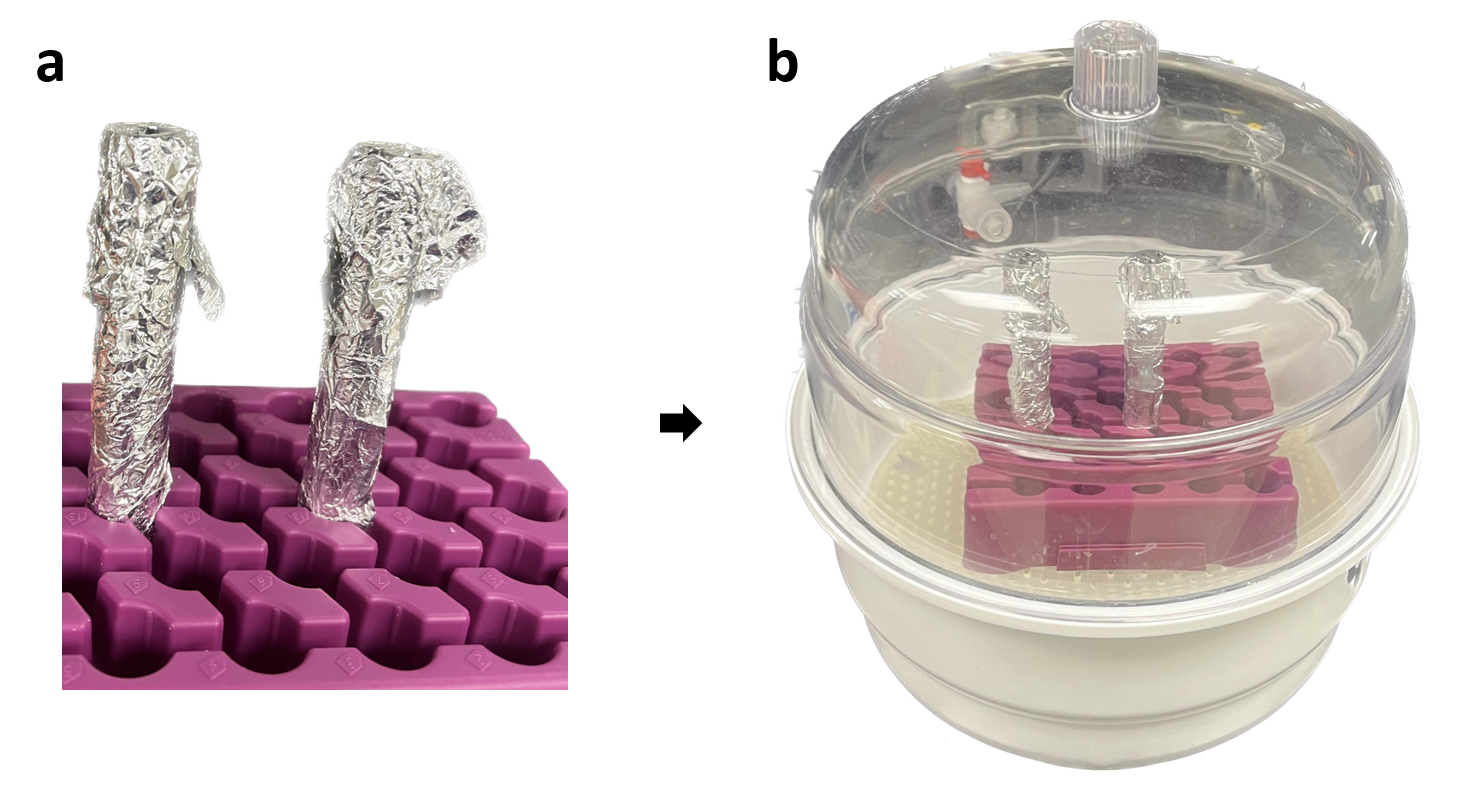


**Figure S5** Pictures showing the infiltration procedure of cotyledon-VIGS. **a** Sprouts or small seedlings were immersed in the infiltration solutions in 15 mL (or 50 mL) tubes. For VIGS of *ChlH* and *PDS*, the tubes were wrapped with aluminum foil. **b** Tubes were placed in the container for vacuum infiltration.

**Table S1 Primers used in this study**

| **primer** | **sequence (5'-3')** |
| --- | --- |
|  | **For RT-qPCR** |
| CrPRS9-qPCR-F | GAGGGCCAAAACAAACTTGA |
| CrRPS9-qPCR-R | CCCTTATGTGCCTTTGCCTA |
| CrChlH-qPCR-F | AGAGAATGCTGGGCTCTACAAG |
| CrChlH-qPCR-R | TTGTCCAAATTACACTGCTTGG |
| CrPDS-qPCR-F | GTGAAGTGCGGCTTAACTCAAG |
| CrPDS-qPCR-R | GCATCTCCTTTGATTGCTGACC |
| T3O-qPCR-F | TTTGCCATTTGGTGCCGGAAGA |
| T3O-qPCR-R | CTGGGAGTTGCCAGTTGAAATGGT |
| T3R-qPCR-F | CGCGAGTACGGGTGGAAGTATAAA |
| T3R-qPCR-R | CGGGGATAACCTCAACATCTGCAA |
| CrGATA1-qPCR-F | CGCCCGATCAAACCCAATTT |
| CrGATA1-qPCR-R | TCCCCTGCAAATCATCATCGT |
| DAT-qPCR-F | ATCGGTTGAGACAGAGACACTCTC |
| DAT-qPCR-R | GATACGCACGTTTGGTATATGTTTT |
| CrMYC2-qPCR-F | TTTGGCAGTCGTCTGTTGTC |
| CrMYC2-qPCR-R | CAAAAGAACTCGCGGAAGAC |
| ORCA2-qPCR-F | TGCGGGAGAACAAGAAGAAG |
| ORCA2-qPCR-R | TTCGATCTCTGCTCACATCC |
| ORCA3-qPCR-F | CGGGATCCGAAATACAGAAA |
| ORCA3-qPCR-R | GCCCTTATACCGGTTCCAAT |
| CrGBF1-qPCR-F | AACAGGCTGAGACGGAAGAA |
| CrGBF1-qPCR-R | GACCCGTGCATTTTTCAACT |
| CrGBF2-qPCR-F | GGAAGGTGCCATCTACTCCA |
| CrGBF2-qPCR-R | CTAGATCGCCGAGCAGATTC |
| GiActin-qPCR-F | TGACCGTATGAGCAAGGAGATC |
| GiActin-qPCR-R | GCTGGAAGGTGCTGAGGGAT |
| GiChlH-qPCR-F | TACATTGTTGCATTGCCACTGG |
| GiChlH-qPCR-R | TTCCATCCCTCCATCTAGCTCA |
| AaActin-qPCR-F | CCAGGCTGTTCAGTCTCTGTAT |
| AaActin-qPCR-R | CGCTCGGTAAGGATCTTCATCA |
| AaChlH-qPCR-F | TGGATTCAGTATTTGGCCGACA |
| AaChlH-qPCR-R | TCTAAAGCTTGTTTCAAGGCGC |
|  | **For VIGS constructs** |
| GiChlH-vigs-F | ACGTGGTACCTCAGCTATCTGACTCCTCC |
| GiChlH-vigs-R | ACGTCTCGAGGATGACATGGAGTCCACAAG |
| AaChlH-vigs-F | ACGTGGTACCGCCAATACCATAAGTTACTTGACTCC |
| AaChlH-vigs-R | ACGTCTCGAGACCGATAACATGAAGACCACA |
| CrGBF1-vigs-F | CAGTGGTACCTAAGTTGAAAAATGCACGGG |
| CrGBF1-vigs-R | TCCATGGGGATACATAGCTGGCATCGAGCAACTG |
| CrGBF2-vigs-F | CAGTTGCTCGATGCCAGCTATGTATCCCCATGGA |
| CrGBF2-vigs-R | TCGACTCGAGCTGCCCAGACTTCCT |
| CrMYC2-vigs-F | TCGAGGTACCCTAGTGTTCAAGGGAATTCAAT |
| CrMYC2-vigs-R | TCGACTCGAGATCCACAACCCTACTACTCT |

**Supplementary Text**

>GiChlH (full length coding sequence from an unpublished transcriptome data)

ATGGCTTCTTTAGTATCATCCCCATTTACTCTATCTAGTTCTAAAGCTGATCAACTTTCTTCCCTTTCTCAAAGACACCTTTTTCTTCATTCTTTCCTTCCAAAGAAGGCCAATTGCTATAACAGCTCAACAAAGTCTTCGCTGAGACCAGTGAAGTGTGCTGCAATTGGGAATGGCCTTTTCACCCAAACCACCCAAGAAGTTCGTAGGATAGTTCCAGAGAACAGCCAAAACCTTCCAACAGTTAAGGTTGTTTATGTTGTCCTTGAAGCTCAGTACCAGTCATCACTCTCAGCTGCTGTTAGAGCCCTCAACAGTACCAACAAAGATGTTTCCTTTGAGGTTGTTGGTTACTTGGTGGAGGAGCTTCGTGATGAGTCCACTTATAAGACTTTTTGCAAGGATTTGGAAGATGCCAACATCTTCATTGGGTCTTTGATCTTTGTGGAGGAGCTAGCACTCAAGGTTAAGGATGCTGTGGAGAAAGAAAGGGACAGGCTTGATGCAGTCCTTGTGTTCCCATCAATGCCTGAGGTAATGAGGCTGAACAAGTTGGGGTCCTTCAGCATGTCACAGCTTGGACAATCCAAGAGCCCCTTTTTCCAGCTCTTCAAAAAGAAGAAGCAGTCTTCTGCTGGCTTTGCTGATAGCATGTTGAAGCTGGTGAGGACTTTGCCTAAGGTTTTGAAGTATTTGCCAAGTGATAAGGCTCAGGATGCTAGACTTTACATCCTGAGCCTGCAGTTTTGGCTTGGTGGATCACCTGATAATCTGCAGAACTTTTTGAAAATGATTTCTGGGTCTTATGTTCCGGCACTGAAAGGGACAAAGATGGAGTATTCGGAACCGGTTTTGTACTTGGACAGTGGGATTTGGCACCCTCTTGCACCTTGTATGTATGATGATGTGAAGGAGTATCTGAATTGGTATGGAACTAGGAGGGATGCTAATGAGAAGCTGAAGAGTCCGAATGCGCCGGTGATCGGTCTGATTTTGCAAAGGAGTCATATTGTTACCGGTGATGAGGGTCATTATGTGGCTGTGATCATGGAACTTGAGGCTAAAGGGGCTAAGGTCATTCCGATTTTCGCCGGGGGGCTTGACTTTTCAGGGCCAGTGGAGAGGTTCTTGATTGATCCAATCACTAAGAAACCGTTTATAAATTCCGTGATATCGCTCACCGGCTTCGCTCTTGTTGGAGGCCCTGCAAGGCAGGACCATCCGAGGGCAGTTGAGGCTTTGATGAAGCTTGATGTTCCTTACATTGTTGCATTGCCACTGGTGTTCCAGACAACAGAAGAGTGGCTGAACAGTACTTTGGGTTTGCATCCAATTCAGGTTGCTCTTCAAGTTGCACTGCCTGAGCTAGATGGAGGGATGGAACCAATTGTTTTTGCTGGTCGTGATCCCAAAACAGGAAAATCTCATGCTCTTCACAAGAGAGTGGAGCAGCTCTGCACCAGGGCAATCAGATGGGCTGAATTGAAAAGAAAGTCAAAGGAAGAGAAGAGACTAGCAATCACTGTCTTCAGTTTCCCACCAGACAAAGGAAATGTAGGAACTGCTGCCTACCTGAACGTCTTCTCCTCCATTTTCTCTGTTTTAAAAGAACTCCAAAGAGATGGTTACAATTGCGAGGGTCTTCCAGAGACTTCAGAAGAATTGATTGAAGAGGTAATACATGACAAAGAAGCGCAATTCAGCAGCCCAAATTTGAATGTTGCTTACAAAATGGGTGTCCGCGAATATCAAAGTCTCACTCCCTATGCCACAGCATTAGAAGAGAATTGGGGAAAGCCTCCTGGGAATCTGAATGCAGATGGAGAGAATCTATTGGTATATGGGAAACAATATGGTAATGTATTCATAGGGGTTCAACCCACATTTGGATATGAAGGCGATCCCATGCGGTTGCTTTTCTCCAAATCTGCAAGCCCACATCATGGTTTTGCAGCATATTACTCTTTTGTTGAGAAAATCTTCAAAGCCGATGCTGTTCTTCATTTCGGGACACACGGTTCCCTTGAATTCATGCCTGGAAAGCAGGTCGGGATGAGTGATGTATGTTACCCTGACAGTCTGATTGGGAATATTCCCAATGTCTATTACTATGCTGCTAACAACCCTTCTGAGGCTACCATAGCCAAACGCAGGAGCTATGCGAATACCATCAGCTATCTGACTCCTCCGGCAGAAAATGCAGGACTGTACAAAGGTCTTAAGCAGTTAAGTGAGCTCATCTCCTCATACCAGTCCCTCAAGGACACTGGCCGTGGGCAACAAATTGTGAGTTCAATTATCAGCACAGCTAAACAGTGTAATCTTGACAAGGATGTGGAACTTCCAGATGAGGGTGTAGAGCTCCCGGCTAAAGAGCGCGACCTTGTTGTCGGGAAGGTGTATTCCAAGATCATGGAGATCGAGTCCCGCCTGTTGCCTTGTGGACTCCATGTCATCGGTGAGCCACCCTCAGCCATGGAAGCAGTTGCTACCCTAGTGAACATAGCTGCACTCGATCGTCCTGAAGACGGTATTTCCTCCCTCCCTTCTATACTAGCTGAGACTGTAGGAAGAAACATAGAGGATGTTTATAGATCTAGTGACAAGGGAATACTAAAGGATGTAGAGCTTCTTCGACAAATAACTGAAGCATCACGCGGAGCAATTACTTCCTTTGTGGAGCGCACTACTAATGATAAGGGTCAAGTTGTTGATGTAAAAGATAAACTAAGCTCGATTCTTGGATTTGGCATAAATGAACCATGGGTTCAGTACTTGTCGAACACCAAATTTTACCGAGCTGATAGGGAAAAACTCAGAACTTTGTTTGAGTTCTTGGGAGAATGTTTGAAGTTGGTCGTAGCTAATAACGAAGTGGGAAGCTTAAAACAAGCCTTGGAGGGTAAATATGTTGAGCCAGGACCTGGAGGTGACCCAATTAGAAACCCTAAAGTTTTACCAACAGGAAAGAATATCCATGCCCTGGACCCACAAGCCATTCCTACTACGGCAGCGATGCAGAGTGCCAAAGTAGTGGTAGAGAGATTGATTGAGAGGCAAAAGGCTGAGAATGGGGGAAAGTATCCTGAGACAATTGCACTTGTACTGTGGGGAACAGATAACATTAAGACATATGGGGAGTCCCTGGCTCAGGTCTTGTGGATGATTGGTGTGAACCCAATAGCCGATACCTTTGGAAGGGTCAACCGGGTAGAACCTGTAAGCCTTGAAGAGCTTGGAAGGCCTAGAATTGATGTCGTCGTCAATTGCTCCGGAGTATTCAGAGACCTTTTTATCAATCAGATGAATCTTCTGGATAGAGCAGTGAAGATGGTTGCTGAATTGGATGAACCCGCAGAGCAGAACTTTGTAAGGAAGCATGCATTGGAACAAGCTCAAGCCCTTGGGATTGAAGTTCGAGAAGCGGCAACAAGGGTCTTCTCCAATGCCTCAGGGTCCTACTCTTCAAACATAAACCTGGCCGTGGAGAATTCTTCATGGAATGACGAGAAGCAGCTCCAGGACATGTACCTTAGCCGTAAGTCTTTCGCTTTTGACTGCGATGCCCCTGGTGCAGGCATGACCGAGAAAAGAAAAGTCTTTGAGATGGCTCTGAGCACAGCAGACGCCACGTTCCAAAACCTCGACTCATCAGAAATCTCCCTCACTGATGTCAGTCATTACTTTGACTCAGACCCAACAAACCTGGTTCAAAATCTAAGGAAAGATGGGAAGAAGCCAAGTGCATACATTGCTGACACAACCACAGCTAATGCCCAGGTACGTACCCTTTCCGAGACCGTCCGACTTGATGCAAGAACCAAGCTGTTGAATCCCAAGTGGTATGAAGGCATGTTGTCTAGTGGCTACGAGGGTGTCCGCGAGATCGAGAAGAGGCTTACCAACACAGTTGGATGGAGTGCAACTTCTGGCCAAGTTGACAACTGGGTGTATGAAGAAGCCAACACAACTTTCATTCAAGATGAGGAGATGCTGAAGAAGCTCATGAACACAAACCCCAACTCCTTCAGGAAGCTGGTGCAGACATTCTTGGAAGCCAACGGGCGCGGATATTGGGAGACTTCGGAACAGAACATTGAGAAACTCAGGCAGTTGTACTCAGAAGTGGAAGACAAAATTGAAGGCATTGATCGTTGA

>AaChlH (gene fragment was assembled from three contigs, comp4310_c0_seq1_1, comp4310_c0_seq9_1 and comp4310_c0_seq5_1, in the transcriptome data from biomedcentral.com/1471-2229/13/220)

ATTTTCGCTGGACGTGACCCAAGAACAGGAAAATCACATGCTCTTCACAAGAGGGTGGAACAGCTATGTACTAGAGCAATCAGATGGGCTGATCTCAAGAGAAAAACAAAGAGTGAGAAGAGGGTGGCGATTACTGTATTCAGTTTCCCACCAGACAAAGGCAATGTTGGAACAGCTGCTTACCTTAATGTATTTGCATCCATTTTCTCTGTTCTACAAGACCTCAAAAGAGATGGTTACAATGTGGAAGGCCTTCCAGCGACGTCCGCAGAGTTGATTGAAGATGTTCTTCACGACAAAGAGGCACAGTTCAGCAGTCCAAATCTGAACGTTGTTTACAAGATGGGAGTGAGAGAGTACCAACAGTTAACTCCATACTCCATAGCATTGGAAGAGAACTGGGGAAAGCCTCCAGGGAACTTGAACTCTGACGGTGAGAACCTTTTGGTTTATGGAAAACAATACGGAAACGTTTTCATTGGTGTCCAGCCTACTTTCGGGTACGAGGGTGATCCCATGAGGCTACTATTCTCTAAATCAGCTAGCCCGCATCACGGATTTGCAGCTTATTACTCTTATGTTGAGAAAATCTTCAAAGCCGATGCGGTTCTCCATTTTGGTACCCACGGGTCATTAGAGTTCATGCCTGGAAAACAGGTCGGGATGAGTGATGCTTGTTTCCCCGACAGTCTTATCGGTAACATCCCAAATGTCTACTATTACGCAGCTAATAATCCTTCAGAAGCCACCATTGCCAAGAGAAGAAGTTACGCCAATACCATAAGTTACTTGACTCCACCTGCTGAAAATGCTGGTCTTTACAAGGGTTTGAAGCAGCTGAGTGAGTTGATTGCATCCTACCAGTCTCTCAAAGATACAGGTCGTGGTCAGCAAATTGTGAGCTCAATCATTAGCACTGCTAAGCAATGCAATCTTGACAAAGATGTAGACCTACCCGAAGAAGGTGTGGAGATTTCAAGCAAAGAACGTGATCTTGTTGTTGGAAAGGTCTACTCCAAGATCATGGAAATCGAGTCTAGACTGTTGCCATGTGGTCTTCATGTTATCGGTGAGCCACCATCAGCCATGGAGGCTGTTGCCACTTTGGTCAACATCGCAGCCTTGGACCGCCCAGAAGAAGGGATTTCGTCTCTTCCATCAATATTGGCTGAGACAGTTGGTAGAGAAATTGAAGATATATACAGAAGCAGTGATAAGGGAATCTTAAAAGATGTCGAGTTGTTGAAGCAAATTACAGATGCGTCCCGTGGTGCTGTTGATGCTTTCGTGCAGAGGAGCACCAACAGTAAGGGTCAAGTGGTTGATATGTCTGGAAAACTAAGTTCAATCCTTGGGTTCGGTCTGAATGAGCCATGGATTCAGTATTTGGCCGACACCAAGTTCTACAGAGCTGATAGGGAGAAGCTTAGAGTTCTATTCCAGTTCTTAGGTGACTGCTTGAAGCTGGTAGTAATGGACAACGAGTTGGGCGCCTTGAAACAAGCTTTAGAAGGTAAGTACGTAGAGCCAGGTCCAGGAGGAGACCCGATTAGAAACCCGAAGGTTTTACCAACCGGAAAGAACATTCACGCATTGGACCCACAAGCAATCCCAACCACAGCTGCAATGCAAAGTGCAATGGTGGTTGTGGACAGGCTGCTCGAGAGGCAAAAGGCTGACAATGGAGGAAAGTTCCCAGAAACAGTTGCATTGGTCTTATGGGGAACTGACAA
